# Peripheral perfusion index combined with end-tidal carbon dioxide in predicting sustained return of spontaneous circulation in adults with in-hospital cardiac arrest: A prospective observational study

**DOI:** 10.1371/journal.pone.0359869

**Published:** 2026-10-28

**Authors:** Hong Zhong, Xianjuan Gou, Nana Xu, Linfei Li, Xiaoling Du, Ya Gong, Zhi Kou, Manhong Zhou

**Affiliations:** 1 Elderly Hip Fracture Treatment Center, Beijing jishuitan hospital Guizhou hospital, Guiyang, China; 2 Emergency Department, Affiliated Hospital of Zunyi Medical University, Zunyi, Guizhou, China; 3 Emergency Department, Kweichow Moutai Group, Zunyi, Guizhou, China; 4 Emergency Department, The Second Affiliated Hospital of Zunyi Medical University, Zunyi, Guizhou, China; Azienda Ospedaliero Universitaria Careggi, ITALY

## Abstract

**Objective:**

To evaluate the associations of peripheral perfusion index (PI) and end-tidal carbon dioxide (P_ET_CO_2_), individually and in combination, with sustained return of spontaneous circulation (ROSC) in adults with in-hospital cardiac arrest, and to assess their predictive performance.

**Methods:**

This prospective observational study included 125 adults with in-hospital cardiac arrest, of whom 62 achieved sustained ROSC and 63 did not. Measurements obtained after ROSC or termination of resuscitation were excluded. Changes in PI and P_ET_CO_2_ during resuscitation were analyzed using linear mixed-effects models. Event-average PI and P_ET_CO_2_ were evaluated for their ability to predict sustained ROSC using receiver operating characteristic (ROC) analysis. The combined model was internally validated using 2,000 bootstrap resamples.

**Results:**

The median time to ROSC was 11 min (IQR, 5–20). Significant group-by-time interactions were observed for both PI and P_ET_CO_2_ (both *P* < 0.001). Event-average PI was higher in the ROSC group than in the non-ROSC group [0.62% (IQR, 0.42–0.90) vs. 0.37% (0.31–0.52)], as was event-average P_ET_CO_2_ [15.5 mmHg (12.2–17.8) vs. 12.0 mmHg (10.4–13.7)] (both *P* < 0.001). The areas under the ROC curve (AUCs) were 0.756 (95% CI, 0.670–0.842) for PI, 0.753 (95% CI, 0.666–0.839) for P_ET_CO_2_, and 0.808 (95% CI, 0.731–0.886) for their combination. The optimism-corrected AUC for the combined model was 0.800. Pairwise comparisons showed no significant differences among the AUCs. In the exploratory fully adjusted model, P_ET_CO_2_ remained independently associated with sustained ROSC (adjusted OR, 1.626; 95% CI, 1.148–2.302; *P* = 0.006), whereas PI was not statistically significant (adjusted OR, 1.359 per 0.1% increase; 95% CI, 0.962–1.920; *P* = 0.082).

**Conclusion:**

Higher PI and P_ET_CO_2_ during resuscitation were associated with sustained ROSC. Combining PI with P_ET_CO_2_ yielded numerically better discrimination than either measure alone, although the differences in AUC were not statistically significant. External validation is needed before the combined model can be applied in clinical practice.

## Introduction

Cardiac arrest (CA), due to its high incidence and mortality rates, has become a major global public health concern. Cardiopulmonary resuscitation (CPR) remains the critical intervention to save the lives of patients experiencing CA [1]. During resuscitation, real-time physiological monitoring may provide useful prognostic and physiological information and complement clinical assessment; several physiological parameters have been shown to be associated with the return of spontaneous circulation (ROSC) [2–4].

Currently, commonly used physiological indicators for predicting ROSC include hemodynamic parameters such as invasive arterial pressure and coronary perfusion pressure (CPP), as well as end-tidal carbon dioxide partial pressure (P_ET_CO_2_) [5,6]. However, the monitoring of hemodynamic indicators often requires additional time, specialized equipment, and skilled personnel, limiting their widespread use during emergency resuscitation. Therefore, identifying a non-invasive, simple, accurate, and real-time method for assessing physiological status during CPR has significant clinical relevance. P_ET_CO_2_ reflects the partial pressure of carbon dioxide in exhaled gas at the end of expiration and has been associated with CPR quality and ROSC in patients with CA [7]. Evidence indicates that, owing to the complex interrelationships among various physiological variables, single-parameter monitoring may have limited predictive accuracy for ROSC. In contrast, conbining multiple indicators may improve the accuracy of prediction [8].

The peripheral perfusion index (PI) is a non-invasive, real-time physiological metric derived from the photoplethysmography signal as the ratio of the pulsatile to non-pulsatile components, reflecting local pulsatility and peripheral perfusion at the sensor site [9]. Recent studies have suggested that PI may be associated with the likelihood of ROSC during CPR [10,11]. In particular, a multicenter prospective study reported that pulse oximetry waveform-derived parameters predicted ROSC in both in-hospital and out-of-hospital cardiac arrest [12], and a recent prospective cohort study found an association between event-average PI and sustained ROSC in out-of-hospital cardiac arrest [13]. However, inconsistencies remain regarding the timing and threshold values of PI measurements during resuscitation, and evidence specifically in patients with IHCA is limited. Hence, the potential value of PI during CPR remains to be further elucidated. Our observational study mainly aimed to investigate the association between PI and ROSC in adults with IHCA. Furthermore, we hypothesized that combining PI with P_ET_CO_2_ for the prediction of ROSC may offer greater clinical utility.

## Materials and methods

### Study population

Adult patients with in-hospital cardiac arrest (IHCA) who were admitted to the Emergency Department or Intensive Care Unit (ICU) of our hospital between January 2023 and June 2024 and received cardiopulmonary resuscitation (CPR) immediately after recognition of cardiac arrest were enrolled in this study. Time zero (T0) was defined as the moment at which chest compressions were initiated, and served as the starting. We included patients with IHCA aged > 18 years who received CPR and underwent tracheal. Exclusion criteria were: (1) out-of-hospital cardiac arrest; (2) traumatic cardiac arrest or arrest due to haemorrhagic or hypovolaemic shock; (3) conditions that could interfere with peripheral oxygen saturation monitoring, such as Raynaud’s phenomenon or vasculitis; (4) pregnancy; (5) absence of tracheal intubation; and (6) incomplete or unreliable PI/P_ET_CO_2_ monitoring data.

### Ethical considerations

PI measurement is a non-invasive monitoring procedure and does not cause harm to study participants. All rescuers received standardized professional training and passed the required assessments. This study was conducted in accordance with the principles of medical ethics. Before the study was initiated, the research protocol was approved by the Biomedical Research Ethics Committee of the Affiliated Hospital of Zunyi Medical University (Approval Number: KLLY-2022–050). Because this was a prospective emergency study conducted during cardiac arrest, a deferred-consent procedure was used: written informed consent was obtained from patients who regained decision-making capacity or from their legal next of kin as soon as feasible after resuscitation. For patients who died before consent could be obtained, the ethics committee granted a waiver of the informed consent requirement. All data were fully anonymized before analysis. This observational study was reported in accordance with the STROBE guidelines.

### Study design

This was a prospective longitudinal study involving adult IHCA patients treated in the Emergency Department and ICU from January 2023 to June 2024.

## Methods

All patients were resuscitated in accordance with the 2020 American Heart Association (AHA) Guidelines for Cardiopulmonary Resuscitation and Emergency Cardiovascular Care [14] and the hospital’s local protocols. Structured reassessment of CPR effectiveness was performed at 5-minute intervals, aligned with the physiological data collection time points; standard rhythm and pulse checks were performed in accordance with the AHA algorithm and were not limited to these intervals. The primary outcome was sustained ROSC, defined as the return of spontaneous circulation maintained for at least 20 minutes without ongoing chest compressions, in accordance with the Utstein definitions [2]. During resuscitation, clinical data were collected using the Mindray BeneView T5 monitor (Mindray Bio-Medical Electronics Co., Ltd., Shenzhen, China) and compatible pulse oximetry sensors (model 512E). Physiological parameters recorded included electrocardiogram (ECG), pulse rate, respiratory rate, perfusion index (PI), and end-tidal carbon dioxide partial pressure (P_ET_CO_2_).

Resuscitation team members (physicians and nurses) in the Emergency Department and ICU received periodic training in high-quality CPR, and additional training was provided on the proper use of capnography and compatible pulse oximetry sensors. Without compromising CPR quality, pulse oximetry sensors were applied to the fingertip as soon as possible after the initiation of CPR, preferably to the middle finger of the hand opposite the intravenous cannula. After successful tracheal intubation, the airway adapter and P_ET_CO_2_ sensor were connected to the patient’s respiratory circuit, and P_ET_CO_2_ was measured continuously.

At each time point, the recorded value was the automatically calculated median of the PI and P_ET_CO_2_ signals over a 10-second window centred on that time point. A value was accepted only when the following prespecified signal-quality criteria were met: a stable and continuous plethysmographic waveform without motion or low-perfusion alarms, a valid perfusion quality index provided by the monitor, and an end-tidal CO_2_ waveform with a stable plateau. If no valid signal was available within the window, the value was classified as missing rather than replaced. Data were extracted independently by two investigators who were blinded to the clinical outcome, and disagreements were resolved by a third investigator.

### Data collection

Data were collected as follows: (1) basic clinical characteristics, including sex, age, initial cardiac rhythm, cause of arrest, and underlying diseases; (2) resuscitation measures, including time to CPR initiation (time from recognition of cardiac arrest by medical staff to initiation of CPR), CPR duration, number of defibrillations, and cumulative dose of epinephrine administered; and (3) PI and P_ET_CO2 values. PI was derived from the photoplethysmographic signal as the ratio of the pulsatile to non-pulsatile components and was expressed as a percentage. PI and P_ET_CO2 were monitored continuously, and values at 0, 5, 10, 15, and 20 minutes after initiation of CPR were extracted automatically from the electronically exported monitor trend data; values were not manually transcribed during resuscitation. Monitoring continued until resuscitation was terminated or sustained ROSC was achieved. Only measurements obtained during ongoing CPR and before or at the assessment resulting in ROSC or termination of resuscitation were included in the analyses; measurements obtained after ROSC or termination were excluded. At each time point, only patients who were still undergoing CPR and had a valid measurement contributed to the analysis. Event-average PI and P_ET_CO_2_ were defined as the arithmetic mean of all available scheduled measurements at 0, 5, 10, 15, and 20 min obtained during active CPR before or at the assessment resulting in ROSC or termination of resuscitation.

### Statistical analysis

All data were analyzed using SPSS version 29.0 (IBM Corp., Armonk, NY, USA) and R software (version 4.3.2; R Foundation for Statistical Computing). All statistical tests were two-sided, with *P* < 0.05 considered statistically significant. Normally distributed continuous variables were summarized as mean ± standard deviation, non-normally distributed variables as median and interquartile range (IQR), and categorical variables as frequencies and percentages. Between-group comparisons used Student's t-test, the Mann-Whitney U test, or the chi-square test as appropriate. The number of patients remaining under active CPR and contributing valid PI and P_ET_CO_2_ measurements was reported at 0, 5, 10, 15, and 20 min. Measurements obtained after ROSC or termination were excluded rather than treated as missing values. Longitudinal PI and P_ET_CO_2_ trajectories were analyzed using linear mixed-effects models with a patient-specific random intercept; fixed effects included categorical time, final ROSC status, and the time-by-ROSC interaction, with the overall interaction assessed by likelihood-ratio testing. For the primary predictive analysis, ROC curves were constructed for event-average PI, event-average P_ET_CO_2_, and a combined binary logistic regression model containing both markers as continuous variables. Optimal cutoffs were determined using the Youden index. AUCs were reported with 95% confidence intervals and compared using the DeLong method, including 95% confidence intervals for AUC differences. The combined model was internally validated using 2,000 bootstrap resamples; apparent and optimism-corrected AUCs and Brier scores were calculated, and calibration was evaluated using a calibration plot, calibration intercept, and calibration slope. A parsimonious multivariable logistic regression model additionally adjusted for the baseline covariates available in the participant-level analytic dataset (age and no-flow time). Multicollinearity was assessed using variance inflation factors, and potential nonlinearity in the logit was examined using x × log(x) terms. As an exploratory sensitivity analysis, landmark ROC analyses were performed at 5, 10, 15, and 20 min among patients who remained under active CPR at each landmark, with subsequent ROSC as the outcome.

## Results

### Baseline characteristics of patients

According to the inclusion and exclusion criteria, 376 adult patients with cardiac arrest were screened during the study period, of whom 240 met the eligibility criteria for IHCA. After excluding patients without tracheal intubation or with incomplete or unreliable PI/P_ET_CO_2_ monitoring data (Fig 1), 125 patients were included in the final analysis: 62 in the ROSC group and 63 in the non-ROSC group. There were no statistically significant differences between the two groups in age, sex, primary diagnosis, or etiology of cardiac arrest. However, the proportion of patients with a shockable initial rhythm was significantly higher in the ROSC group than in the non-ROSC group (35.5% vs. 17.5%, *P* < 0.05), as shown in Table 1.

**Table 1:** Comparison of general data between ROSC group and non-ROSC group.

Variables	ROSC (n = 62)	Non-ROSC (n = 63)	*P*-value
Age (years), mean±SD	59.1 ± 14.7	61.7 ± 14.2	0.325
Sex, n (%)			0.792
Male	32 (51.6)	34 (54.0)	
Female	30 (48.4)	29 (46.0)	
Diagnosis, n (%)			
Circulatory system diseases			
Acute inferior myocardial infarction	7 (11.3)	6 (9.5)	0.746
Lethal arrhythmias	5 (8.1)	5 (7.9)	1.000
Heart failure	5 (8.1)	4 (6.3)	0.980
Others	4 (6.5)	5 (7.9)	1.000
Respiratory system disease			
Respiratory Failure	9 (14.5)	5 (7.9)	0.244
Severe pneumonia	6 (9.7)	10 (15.9)	0.300
Others	3 (4.8)	2 (3.2)	0.985
Nervous disease			
Hypoxic brain injury	1 (1.6)	3 (4.8)	0.623
Cerebral infarction / Cerebral hemorrhage	10 (16.1)	9 (14.3)	0.774
Other diagnosis			
Gastrointestinal bleeding	3 (4.8)	6 (9.5)	0.505
Diabetic ketoacidosis	2 (3.2)	3 (4.8)	1.000
Others	7 (11.3)	5 (7.9)	0.525
Cause of cardiac arrest, n (%)			0.513
Cardiac disease	19 (30.6)	16 (25.4)	
Non-cardiac disease	43 (69.4)	47 (74.6)	
Initial arrest rhythm, n (%)			0.022
Shockable cardiac rhythm	22 (35.5)	11 (17.5)	
Non-shockable cardiac rhythm	40 (64.5)	52 (82.5)	

Medical diagnosis was abstracted from the uploaded medical-record-based basic-characteristics file. Cause of cardiac arrest was classified as cardiac or non-cardiac based on the documented medical diagnosis.

Data are presented as mean ± SD or n (%), as appropriate. P-values were calculated for comparisons between the ROSC and non-ROSC groups.

Abbreviations: ROSC, return of spontaneous circulation; SD, standard deviation.

**Fig 1:**
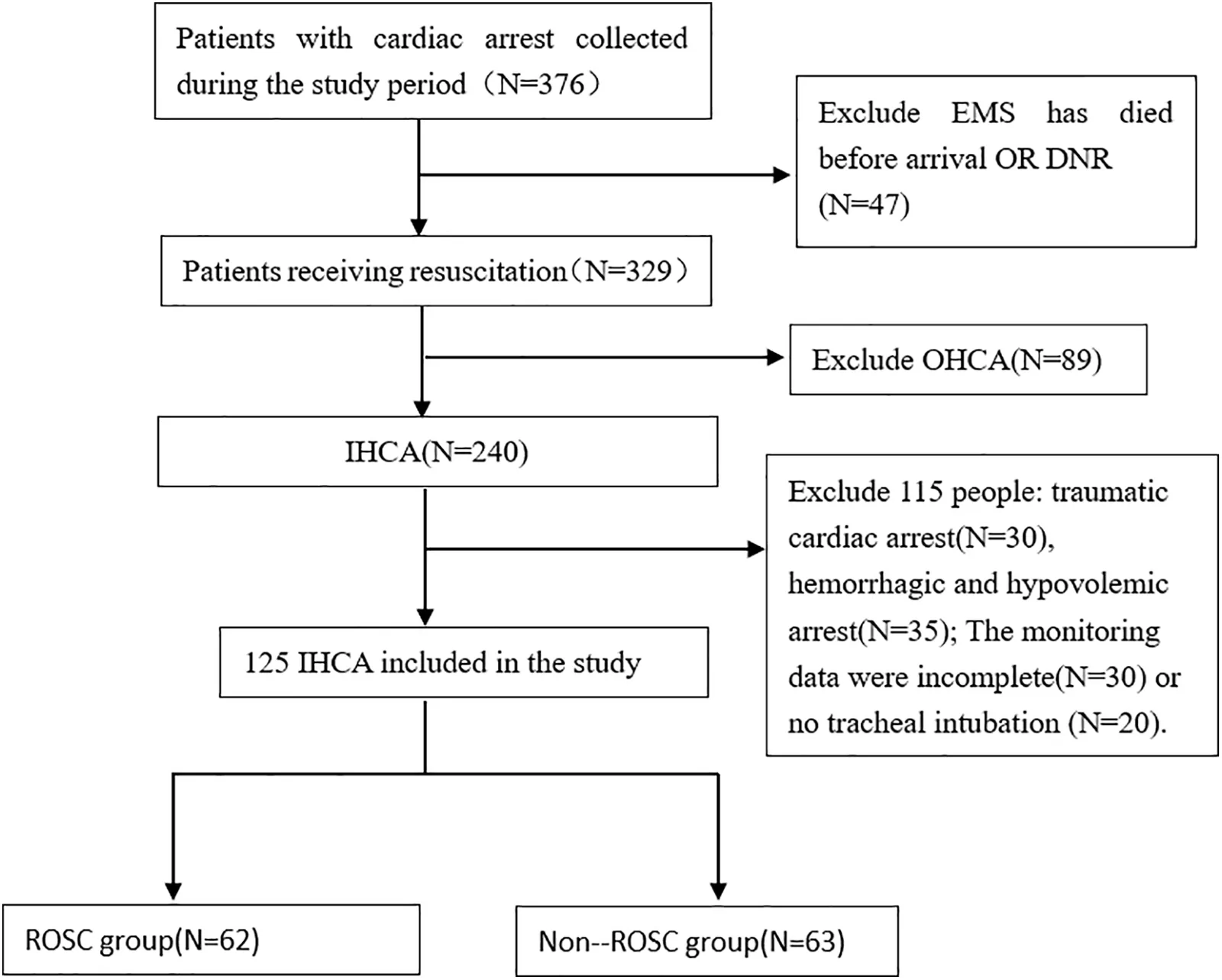
The study flowchart.

### Comparison of resuscitation measures between the ROSC and Non-ROSC Groups

In terms of resuscitation measures, patients in the ROSC group had a shorter time to CPR initiation, shorter overall duration of CPR, lower cumulative dose of epinephrine, and less frequent use of 5% sodium bicarbonate compared to those in the non-ROSC group. Additionally, the proportion of patients who underwent electrical defibrillation was significantly higher in the ROSC group than in the non-ROSC group (38.7% vs. 20.6%, *P* = 0.027), as shown in Table 2.

**Table 2:** Comparison of rescue measures between the two groups.

Rescue measures	ROSC (N = 62)	Non-ROSC (N = 63)	*P*-value
CPR initial time (min), M (Q_L_, Q_U_)	2 (2,2)	2 (2,9)	<0.001
Defibrillation			0.027
Yes	24 (38.7)	13 (20.6)	
No	38 (61.3)	50 (79.4)	
Epinephrine repeated dose			<0.001
≤5 mg	47 (75.8)	10 (15.9)	
> 5 mg	15 (24.2)	53 (84.1)	
CPR duration			<0.001
≤30 min	59 (95.2)	18 (28.6)	
> 30 min	3 (4.8)	45 (71.4)	
5% sodium bicarbonate dose (ml), M (Q_L_, Q_U_)	125 (125,250)	250 (125,250)	0.016

Note: Data are presented as median (IQR) or n (%), as appropriate. *P*-values were calculated for comparisons between the ROSC and non-ROSC groups.

Abbreviations: ROSC, return of spontaneous circulation; CPR, cardiopulmonary resuscitation; IQR, interquartile range.

### Longitudinal changes in PI and P_ET_CO_2_ during active resuscitation

Among the 62 patients who achieved sustained ROSC, the median time from CPR initiation to ROSC was 11 min (IQR, 5–20 min; range, 1–56 min). After excluding measurements obtained after ROSC or termination of resuscitation, the numbers of patients contributing valid measurements at 0, 5, 10, 15, and 20 min were 62, 48, 34, 28, and 17 in the ROSC group and 63, 63, 62, 62, and 62 in the non-ROSC group, respectively. Among patients who were still undergoing active CPR at each scheduled assessment, no additional PI or P_ET_CO_2_ measurements were missing. Linear mixed-effects models showed significant group-by-time interactions for both PI (likelihood-ratio χ² = 56.92, df = 4, *P* < 0.001) and P_ET_CO_2_ (χ² = 64.31, df = 4, *P* < 0.001). Event-average PI was higher in the ROSC group than in the non-ROSC group [0.62% (IQR, 0.42–0.90) vs. 0.37% (IQR, 0.31–0.52); *P* < 0.001]. Similarly, event-average PETCO_2_ was higher in the ROSC group than in the non-ROSC group [15.5 mmHg (IQR, 12.2–17.8) vs. 12.0 mmHg (IQR, 10.4–13.7); *P* < 0.001], as shown in Table 3.

**Table 3:** PI and P_ET_CO_2_ measurements obtained during active CPR, with numbers of contributing participants.

Parameter	Time	ROSC (N)	ROSC median (IQR)	Non-ROSC (N)	Non-ROSC median (IQR)	*P*-value
PI (%)	0 min	62	0.62 (0.27–0.79)	63	0.58 (0.28–0.76)	0.894
PI (%)	5 min	48	0.74 (0.40–1.00)	63	0.31 (0.16–0.78)	<0.001
PI (%)	10 min	34	0.75 (0.47–1.08)	62	0.42 (0.25–0.51)	<0.001
PI (%)	15 min	28	0.91 (0.54–1.47)	62	0.31 (0.21–0.51)	<0.001
PI (%)	20 min	17	1.02 (0.48–1.68)	62	0.18 (0.12–0.38)	<0.001
PI (%)	Event-average	62	0.62 (0.42–0.90)	63	0.37 (0.31–0.52)	<0.001
P_ET_CO_2_ (mmHg)	0 min	62	12.0 (9.0–15.0)	63	10.0 (8.0–12.0)	0.010
P_ET_CO_2_ (mmHg)	5 min	48	15.0 (12.0–18.0)	63	12.0 (11.0–14.0)	<0.001
P_ET_CO_2_ (mmHg)	10 min	34	19.0 (16.0–22.0)	62	14.0 (12.0–17.0)	<0.001
P_ET_CO_2_ (mmHg)	15 min	28	20.0 (14.0–27.0)	62	12.0 (10.0–13.0)	<0.001
P_ET_CO_2_ (mmHg)	20 min	17	26.0 (15.0–30.0)	62	10.0 (7.0–12.0)	<0.001
P_ET_CO_2_ (mmHg)	Event-average	62	15.5 (12.2–17.8)	63	12.0 (10.4–13.7)	<0.001

Data are presented as median (IQR). PI is expressed as % and P_ET_CO_2_ as mmHg. At 0, 5, 10, 15, and 20 min, the numbers of available observations were 62, 48, 34, 28, and 17 in the ROSC group and 63, 63, 62, 62, and 62 in the non-ROSC group, respectively. Event-average values were calculated as the arithmetic mean of all valid scheduled measurements obtained during active CPR; measurements after ROSC or termination were excluded.

Abbreviations: PI, peripheral perfusion index; P_ET_CO_2_, end-tidal carbon dioxide; CPR, cardiopulmonary resuscitation; ROSC, return of spontaneous circulation; IQR, interquartile range.

### Predictive Value of Event-Average PI and P_ET_CO_2_ for Sustained ROSC

Event-average PI yielded an AUC of 0.756 (95% CI, 0.670–0.842), with an optimal cutoff of 0.593%, a sensitivity of 56.5%, and a specificity of 87.3%. Event-average P_ET_CO_2_ yielded an AUC of 0.753 (95% CI, 0.666–0.839), with an optimal cutoff of 15.5 mmHg, a sensitivity of 51.6%, and a specificity of 95.2%. The combined logistic model was defined as logit[P(ROSC)] = −5.423 + 2.678 × event-average PI + 0.296 × event-average P_ET_CO_2_. The combined model yielded an apparent AUC of 0.808 (95% CI, 0.731–0.886); the optimal predicted-probability threshold was 0.572, with a sensitivity of 62.9% and specificity of 90.5% (Fig 2 and Table 4).

**Table 4:** Predictive performance of event-average PI, event-average P_ET_CO_2_, and their combined model.

Predictor	AUC	95% CI	Optimal cutoff	Sensitivity (%)	Specificity (%)
Event-average PI	0.756	0.670–0.842	0.593%	56.5	87.3
Event-average P_ET_CO_2_	0.753	0.666–0.839	15.5 mmHg	51.6	95.2
PI + P_ET_CO_2_	0.808	0.731–0.886	Probability 0.572	62.9	90.5

Event-average values were calculated from valid scheduled measurements obtained during active CPR, with post-ROSC/termination measurements excluded. The combined model included event-average PI and P_ET_CO_2_ as continuous variables. Optimal cutoffs were determined using the Youden index.

Abbreviations: PI, peripheral perfusion index; P_ET_CO_2_, end-tidal carbon dioxide; ROSC, return of spontaneous circulation; AUC, area under the receiver operating characteristic curve; CI, confidence interval.

**Fig 2:**
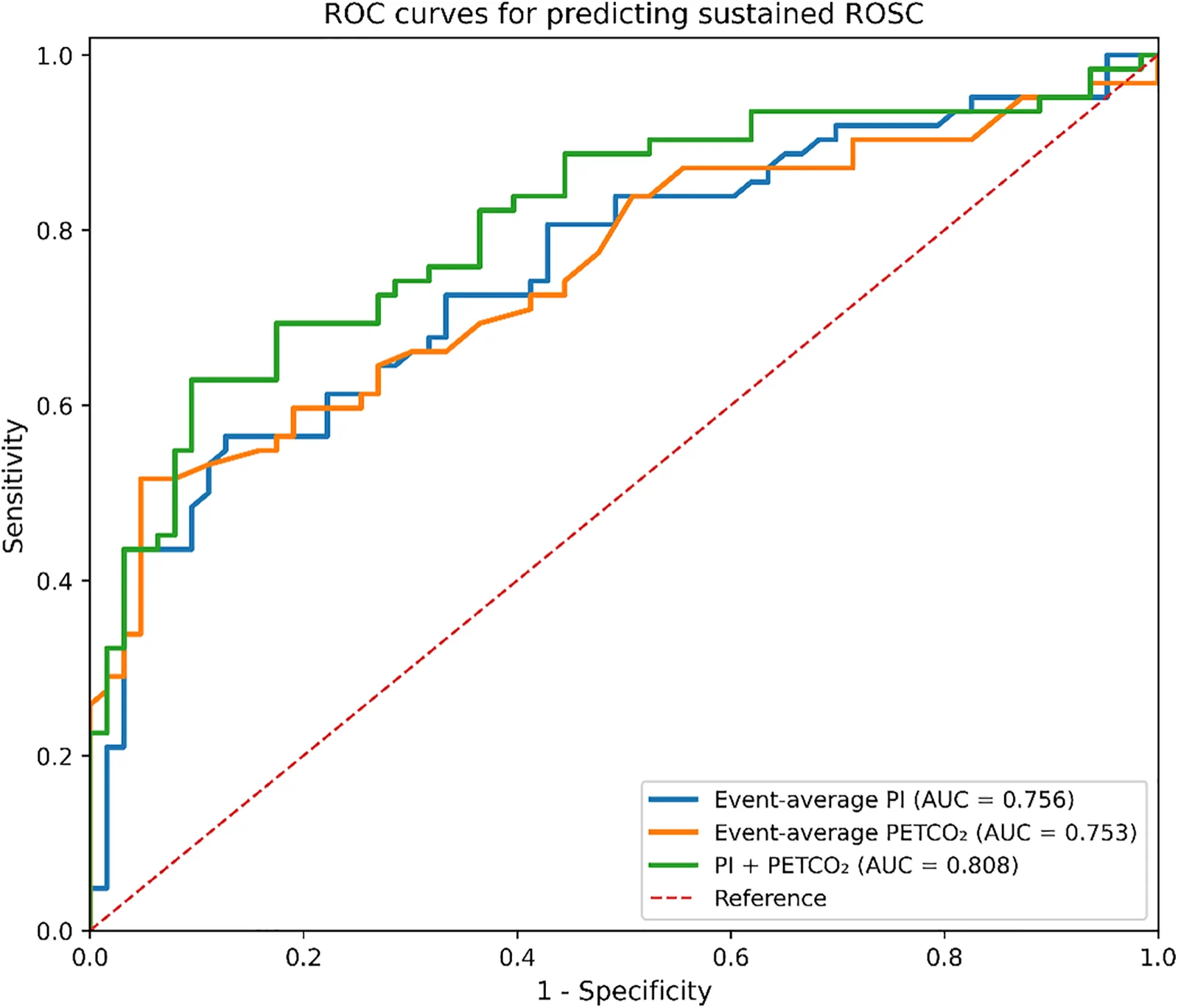
ROC curves for event-average PI, event-average P_ET_CO_2_, and their combined model for predicting sustained ROSC.

### Comparison, internal validation, and calibration of the combined model

DeLong comparisons showed no statistically significant difference between event-average PI and P_ET_CO_2_ (ΔAUC = 0.003, 95% CI, −0.107 to 0.113; *P* = 0.954). The combined model had a numerically higher AUC than PI alone (ΔAUC = 0.053, 95% CI, −0.018 to 0.123; *P* = 0.146) and P_ET_CO_2_ alone (ΔAUC = 0.056, 95% CI, −0.004 to 0.115; *P* = 0.066), but neither difference reached statistical significance. After 2,000 bootstrap resamples, the optimism-corrected AUC was 0.800, the optimism-corrected Brier score was 0.187, and the bootstrap-corrected calibration intercept and slope were −0.008 and 0.931, respectively (Table 5 and Fig 3).

**Table 5:** DeLong comparisons and bootstrap internal validation of the combined model.

Analysis	Estimate	95% CI	*P*-value
PI vs P_ET_CO_2_, ΔAUC	0.003	−0.107 to 0.113	0.954
PI vs combined, ΔAUC	−0.053	−0.123 to 0.018	0.146
P_ET_CO_2_ vs combined, ΔAUC	−0.056	−0.115 to 0.004	0.066
Apparent AUC	0.808		
Optimism-corrected AUC	0.800		
Optimism-corrected Brier score	0.187		
Calibration intercept	−0.008		
Calibration slope	0.931		

Pairwise AUC comparisons were performed using the DeLong method. Internal validation of the combined model was performed using 2,000 bootstrap resamples. Corrected estimates represent optimism-corrected model performance.

Abbreviations: PI, peripheral perfusion index; P_ET_CO_2_, end-tidal carbon dioxide; AUC, area under the receiver operating characteristic curve; CI, confidence interval.

**Fig 3:**
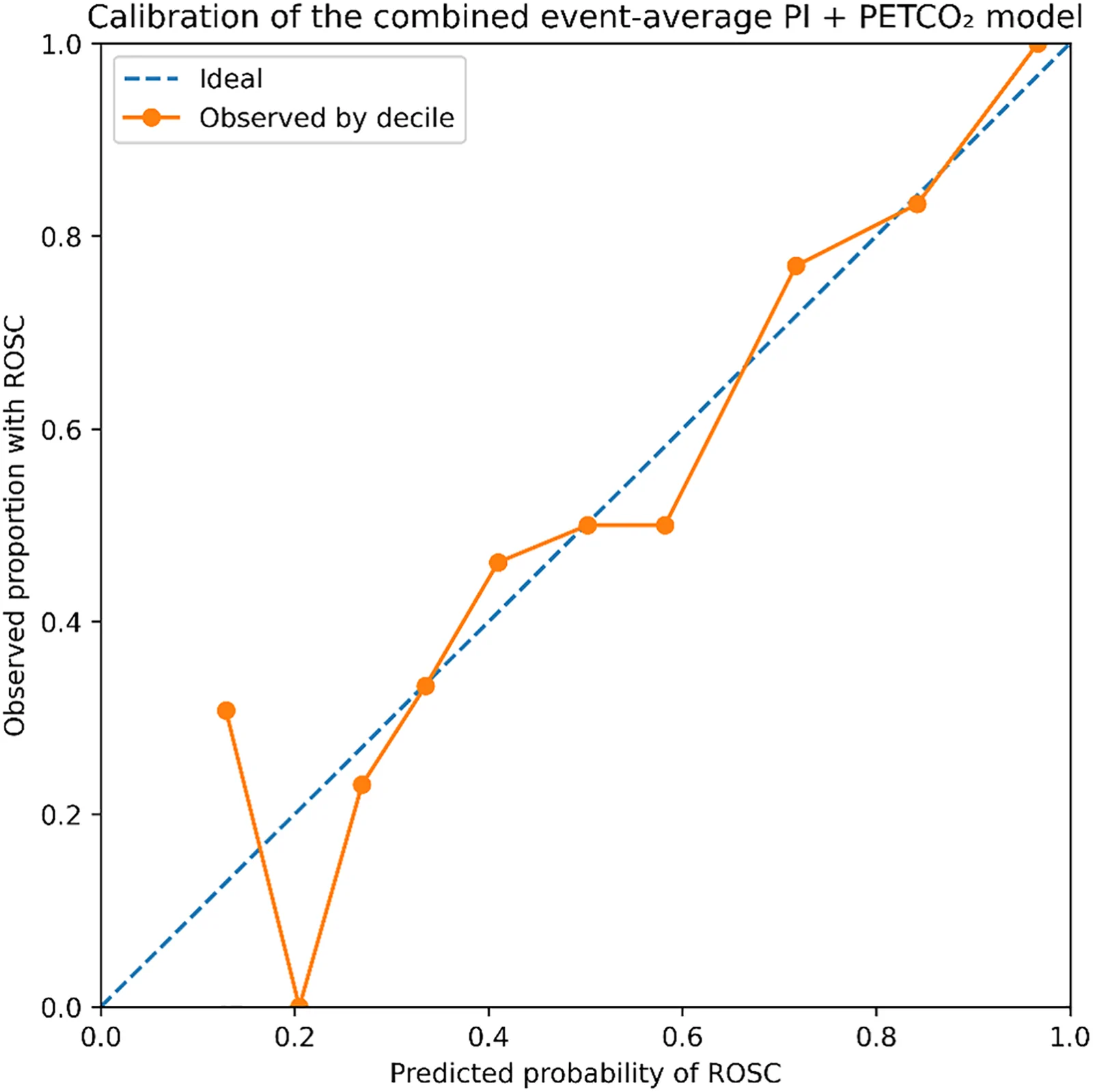
Calibration plot for the combined event-average PI + P_ET_CO_2_ model.

### Multivariable and landmark sensitivity analyses

In the fully adjusted multivariable logistic regression model, all available baseline and resuscitation-related variables were entered simultaneously. Longer no-flow time remained associated with a lower likelihood of sustained ROSC (adjusted OR, 0.670 per minute; 95% CI, 0.461–0.975; *P* = 0.037). Higher event-average P_ET_CO_2_ was associated with a greater likelihood of sustained ROSC (adjusted OR, 1.626 per 1-mmHg increase; 95% CI, 1.148–2.302; *P* = 0.006). An epinephrine dose ≤5 mg (vs. > 5 mg) and CPR duration ≤30 min (vs. > 30 min) were also associated with a greater likelihood of sustained ROSC (adjusted OR, 10.022; 95% CI, 2.052–48.944; *P* = 0.004 and adjusted OR, 9.829; 95% CI, 1.628–59.340; P = 0.013, respectively).

After simultaneous adjustment for all variables, event-average PI showed a positive association with sustained ROSC, although the association did not reach statistical significance (adjusted OR, 1.359 per 0.1% increase; 95% CI, 0.962–1.920; *P* = 0.082). Age, time to CPR initiation, initial rhythm, converted rhythm, sodium bicarbonate administration, and defibrillation were not significantly associated with sustained ROSC in the fully adjusted model (all *P* > 0.05). No substantial multicollinearity was identified, with all variance inflation factors (VIFs) <3 (Table 6).

**Table 6:** Multivariable logistic regression analysis of factors associated with sustained ROSC.

Variable	β	SE	Adjusted OR	95% CI	*P*-value
Age, per year	0.027	0.031	1.028	0.967–1.092	0.382
Time to CPR initiation, per min	0.105	0.222	1.111	0.720–1.716	0.634
No-flow time, per min	−0.400	0.191	0.67	0.461–0.975	0.037
Average PI, per 0.1% increase	0.307	0.176	1.359	0.962–1.920	0.082
Average P_ET_CO_2_, per 1 mmHg increase	0.486	0.177	1.626	1.148–2.302	0.006
Epinephrine dose ≤5 mg vs > 5 mg	2.305	0.809	10.022	2.052–48.944	0.004
CPR duration ≤30 min vs > 30 min	2.285	0.917	9.829	1.628–59.340	0.013
Initial rhythm, code 1 vs 2	0.64	1.091	1.897	0.224–16.091	0.557
Converted rhythm, code 1 vs 2	0.39	1.156	1.476	0.153–14.225	0.736
Sodium bicarbonate, per 125 mL	0.058	0.212	1.059	0.699–1.605	0.786
Defibrillation, code 1 vs 2	0.105	1.117	1.111	0.124–9.917	0.925

Adjusted ORs were estimated using multivariable logistic regression. Event-average PI and P_ET_CO_2_ were modeled as continuous variables; ORs for PI and sodium bicarbonate are reported per 0.1% and 125-mL increase, respectively. Reference categories for categorical variables are indicated in the table.

Abbreviations: ROSC, return of spontaneous circulation; PI, peripheral perfusion index; P_ET_CO_2_, end-tidal carbon dioxide; CPR, cardiopulmonary resuscitation; OR, odds ratio; CI, confidence interval.

## Discussion

### Association between the perfusion index (PI) and Sustained ROSC

The goal of cardiopulmonary resuscitation (CPR) is not only to temporarily restore circulatory perfusion to vital organs such as the heart and brain, but also to achieve return of spontaneous circulation (ROSC), which is regarded as a key clinical outcome of CPR [15]. Evidence suggests that patients with better peripheral perfusion during CPR have a higher likelihood of achieving ROSC [9]. In the present cohort study, PI values recorded during active CPR were significantly higher in patients who achieved sustained ROSC than in those who did not (Table 3). This association is consistent with a multicenter observational study in which pulse-oximetry-derived parameters predicted ROSC in cardiac arrest [16], and with a prospective cohort study reporting that higher event-average PI during out-of-hospital CPR was associated with sustained ROSC, with an event-average PI of 0.614% proposed as a possible threshold [13]. Because no direct measures of CPR quality (compression depth, compression rate, chest-compression fraction, recoil, peri-shock pauses, or ventilation parameters) were collected, PI should not be interpreted as a direct measure of CPR quality, and any role of PI in guiding resuscitation remains to be established.

Following cardiac arrest (CA), a cessation of effective cardiac output that causes a rapid fall in systemic blood flow, perfusion pressure, and peripheral pulsatility, resulting in a low or absent peripheral perfusion state. Therefore, during the initial phase of CPR, there was no significant difference in PI between the ROSC and non-ROSC groups. However, with continued high-quality CPR, systemic circulation gradually improves, blood perfusion to critical organs such as the heart and brain begins to recover, and microcirculatory perfusion is progressively reestablished. Once perfusion is restored to a sufficient extent, it may result in the return of spontaneous circulation (ROSC). Our findings showed that PI values in the ROSC group increased progressively over the course of resuscitation, while the PI values in the non-ROSC group remained consistently low or even declined. A significant difference in PI values between the two groups was observed as early as 5 minutes into CPR, with higher values noted in the ROSC group. A similar trajectory was observed for P_ET_CO2 (Table 3), consistent with reports from Chinese emergency departments in which P_ET_CO2 measured during CPR reflected the quality of resuscitation and was associated with ROSC and better prognosis [17]. These trajectory patterns also support the concept that combined physiological monitoring, such as the simultaneous use of capnography and pulse oximetry, may help identify patients at risk of unsustained ROSC [18], and that perfusion-related parameters are related to the prognosis of resuscitated patients [19]. Whether PI can guide adjustments to compression rate or depth, or detect recurrent cardiac arrest, remains a hypothesis that requires prospective interventional evaluation. If the PI remains persistently low or continues to decline despite ongoing high-quality CPR, it indicates that adequate peripheral perfusion has not been established and the likelihood of ROSC is low. The optimal duration of CPR remains uncertain and depends on multiple patient- and resuscitation-related factors; decisions about termination of resuscitation should be based on established multimodal prognostication within the applicable clinical and ethical framework.

### Predictive value of event-average PI and P_ET_CO_2_

P_ET_CO_2_ is an established physiological marker during CPR and has been associated with generated pulmonary blood flow and ROSC [14,20–23]. Because PI and P_ET_CO_2_ reflect different aspects of the circulation, combining them may provide complementary information. In the present study, event-average PI and P_ET_CO_2_ showed similar discrimination (AUCs 0.756 and 0.753), whereas their combination yielded an apparent AUC of 0.808. However, DeLong comparisons did not demonstrate a statistically significant improvement of the combined model over either marker alone.

The AUCs observed here are comparable with those reported in recent studies of PI and P_ET_CO2 during cardiac arrest [10,13] and in Chinese cohorts using combined capnography and pulse-oximetry monitoring [18,19]. The optimal event-average cutoffs in the present study (PI 0.593%; P_ET_CO2 15.5 mmHg) are also consistent with the event-average PI threshold of 0.614% proposed by Malinverni et al. [13]. Given the limited sensitivities of these cutoffs (56.5% and 51.6%), the single-centre origin of the data, and the absence of external validation, these thresholds should be regarded as exploratory and must not be used as isolated criteria for terminating resuscitation. Accordingly, the present findings support only a numerical improvement in discrimination rather than evidence of statistical superiority.

The combined model was internally validated with 2,000 bootstrap resamples. Its optimism-corrected AUC was 0.800 compared with an apparent AUC of 0.808, with a bootstrap-corrected calibration slope of 0.931 and an optimism-corrected Brier score of 0.187. These results suggest limited apparent overfitting within this cohort, but internal validation cannot substitute for evaluation in an independent population. In the fully adjusted model incorporating both baseline and resuscitation-related variables, average P_ET_CO_2_ remained significantly associated with sustained ROSC, whereas the positive association between average PI and ROSC was attenuated and no longer reached conventional statistical significance (adjusted OR, 1.359 per 0.1% increase; 95% CI, 0.962–1.920; P = 0.082). This attenuation should be interpreted cautiously because several variables included in the fully adjusted model, particularly CPR duration and cumulative epinephrine dose, are time-dependent resuscitation characteristics that may themselves be influenced by the occurrence and timing of ROSC. Landmark analyses were also performed to preserve the temporal ordering between predictors and outcome. Among patients still under active CPR, the combined AUC increased from 0.764 at 5 min to 0.853 at 20 min. Nevertheless, only 17 patients subsequently achieved ROSC among the 79 patients eligible at the 20-min landmark. This small event count and the changing risk set make later landmark estimates unstable and selected. Therefore, neither the 20-min PI value nor any cutoff derived from this single-center cohort should be used as an isolated criterion for termination of resuscitation.

This study has several limitations. First, it was a single-center prospective observational study with a modest sample size, and exclusion of a substantial proportion of screened cardiac-arrest cases may introduce selection bias. Second, the number of patients remaining under active CPR decreased over time, particularly among patients who ultimately achieved ROSC, limiting the precision and generalizability of later landmark analyses. Third, PI can be affected by vascular tone, vasoactive medications, temperature, anemia, sensor position, motion, and other factors that were not fully controlled. Fourth, the multivariable model included a limited number of predictors relative to the number of events, and residual confounding by unmeasured factors cannot be excluded. Fifth, the combined prediction model was internally validated but has not undergone external validation. Finally, this study evaluated sustained ROSC rather than neurologically favorable survival or long-term outcome. Future multicenter studies with larger samples, standardized measurement procedures, comprehensive confounder assessment, and external validation are required.

## Conclusion

In summary, higher peripheral perfusion index (PI) and P_ET_CO_2_ values measured during active CPR were associated with sustained ROSC in adults with in-hospital cardiac arrest. Event-average PI and P_ET_CO_2_ showed moderate discrimination, and their combination showed numerically higher discrimination than either measure alone. These findings support further investigation of PI as an adjunctive physiological marker during resuscitation, but PI should not be considered a direct measure of CPR quality or used as an isolated criterion for terminating resuscitation. External validation is required before clinical use of the combined model or its derived thresholds.

## Supporting information

S1 Text S1 File. Dataset (Excel). S2 File. Data dictionary (Word).
(ZIP)
