# Supplementary material for: Peripheral perfusion index combined with end-tidal carbon dioxide in predicting sustained return of spontaneous circulation in adults with in-hospital cardiac arrest: A prospective observational study
Source: PLoS One. 2026 Oct 28;21(10):e0359869. doi: 10.1371/journal.pone.0359869 (PMC13716206; doi:10.1371/journal.pone.0359869)
Supplement: Supplementary file 1 [file pone.0359869.s001.zip › Supporting Information/S2 Code/Core R Code for the Statistical analysis.docx]

Core R Code for the Statistical analysis

**1. Packages and data import**

\# Install once if required:

\# install.packages(c("readxl","dplyr","tidyr","lme4","pROC",

\#	"boot","ggplot2","car","broom"))

library(readxl)

library(dplyr)

library(tidyr)

library(lme4)

library(pROC)

library(boot)

library(ggplot2)

library(car)

library(broom)

file_path <- "ROSC_analysis_dataset_English.xlsx"

raw <- read_excel(file_path, sheet = "Raw_Data_English")

dat <- read_excel(file_path, sheet = "Analysis_Data")

long <- read_excel(file_path, sheet = "Long_Data")

dat$sustained_rosc <- as.integer(dat$sustained_rosc)

**2. Coding of categorical variables**

dat <- dat %>%

mutate(

rosc_factor = factor(sustained_rosc,

levels = c(0, 1),

labels = c("Non-ROSC", "ROSC")),

epinephrine_dose_group = factor(

epinephrine_dose_group,

levels = c(2, 1),

labels = c(">5 mg", "<=5 mg")

),

cpr_duration_group = factor(

cpr_duration_group,

levels = c(2, 1),

labels = c(">30 min", "<=30 min")

),

initial_rhythm = factor(

initial_rhythm_code,

levels = c(2, 1),

labels = c("Non-shockable", "Shockable")

),

defibrillation = factor(

defibrillation_code,

levels = c(2, 1),

labels = c("No", "Yes")

)

)

**3. Temporal validity rule and post-event exclusion**

The scheduled measurement at time t is retained when t <= CPR duration, because the study team clarified that the scheduled PI/PETCO2 measurement was obtained immediately before the corresponding assessment for ROSC or termination. Measurements at later scheduled time points are excluded.

times <- c(0, 5, 10, 15, 20)

\# Reconstruct long-format data directly from the analysis-ready wide file

long2 <- dat %>%

select(

patient_id, sustained_rosc, cpr_duration_min,

pi_0min_pct, pi_5min_pct, pi_10min_pct, pi_15min_pct, pi_20min_pct,

petco2_0min_mmhg, petco2_5min_mmhg, petco2_10min_mmhg,

petco2_15min_mmhg, petco2_20min_mmhg

) %>%

pivot_longer(

cols = matches("^(pi|petco2)_"),

names_to = c(".value", "time"),

names_pattern = "(pi|petco2)_(\\d+)min.*"

) %>%

mutate(

time_min = as.numeric(time),

measurement_valid = time_min <= cpr_duration_min,

pi = ifelse(measurement_valid, pi, NA_real_),

petco2 = ifelse(measurement_valid, petco2, NA_real_)

) %>%

select(patient_id, sustained_rosc, cpr_duration_min,

time_min, pi, petco2, measurement_valid)

\# Only active-CPR measurements for longitudinal analyses

long_valid <- long2 %>%

filter(measurement_valid)

**4. Time to ROSC and numbers contributing at each time point**

\# Time to ROSC among patients with sustained ROSC

rosc_time_summary <- dat %>%

filter(sustained_rosc == 1) %>%

summarise(

n = n(),

median_min = median(cpr_duration_min, na.rm = TRUE),

q1_min = quantile(cpr_duration_min, 0.25, na.rm = TRUE),

q3_min = quantile(cpr_duration_min, 0.75, na.rm = TRUE),

min_min = min(cpr_duration_min, na.rm = TRUE),

max_min = max(cpr_duration_min, na.rm = TRUE)

)

rosc_time_summary

\# Number at risk / contributing valid measurements

contributing_n <- long2 %>%

filter(measurement_valid) %>%

group_by(time_min, sustained_rosc) %>%

summarise(

n_under_cpr = n(),

pi_available = sum(!is.na(pi)),

pi_missing = sum(is.na(pi)),

petco2_available = sum(!is.na(petco2)),

petco2_missing = sum(is.na(petco2)),

.groups = "drop"

)

contributing_n

**5. Revised Table 3: median (IQR) at each scheduled time**

summary_median_iqr <- function(x) {

x <- x[!is.na(x)]

c(

n = length(x),

median = median(x),

q1 = quantile(x, 0.25),

q3 = quantile(x, 0.75)

)

}

table3_pi <- long_valid %>%

group_by(time_min, sustained_rosc) %>%

summarise(

n = sum(!is.na(pi)),

median = median(pi, na.rm = TRUE),

q1 = quantile(pi, 0.25, na.rm = TRUE),

q3 = quantile(pi, 0.75, na.rm = TRUE),

.groups = "drop"

)

table3_pet <- long_valid %>%

group_by(time_min, sustained_rosc) %>%

summarise(

n = sum(!is.na(petco2)),

median = median(petco2, na.rm = TRUE),

q1 = quantile(petco2, 0.25, na.rm = TRUE),

q3 = quantile(petco2, 0.75, na.rm = TRUE),

.groups = "drop"

)

\# Descriptive between-group P values at each time point

p_pi <- long_valid %>%

group_by(time_min) %>%

summarise(

p_value = wilcox.test(pi ~ sustained_rosc, exact = FALSE)$p.value

)

p_pet <- long_valid %>%

group_by(time_min) %>%

summarise(

p_value = wilcox.test(petco2 ~ sustained_rosc, exact = FALSE)$p.value

)

**6. Longitudinal linear mixed-effects models**

long_valid <- long_valid %>%

mutate(

patient_id = factor(patient_id),

sustained_rosc = factor(sustained_rosc, levels = c(0, 1)),

time_factor = factor(time_min, levels = c(0, 5, 10, 15, 20))

)

\# PI model

fit_pi_full <- lmer(

pi ~ time_factor * sustained_rosc + (1 | patient_id),

data = long_valid,

REML = FALSE

)

fit_pi_no_int <- lmer(

pi ~ time_factor + sustained_rosc + (1 | patient_id),

data = long_valid,

REML = FALSE

)

anova(fit_pi_no_int, fit_pi_full)	# overall group x time interaction

summary(fit_pi_full)

\# PETCO2 model

fit_pet_full <- lmer(

petco2 ~ time_factor * sustained_rosc + (1 | patient_id),

data = long_valid,

REML = FALSE

)

fit_pet_no_int <- lmer(

petco2 ~ time_factor + sustained_rosc + (1 | patient_id),

data = long_valid,

REML = FALSE

)

anova(fit_pet_no_int, fit_pet_full)

summary(fit_pet_full)

**7. Event-average PI and PETCO2**

event_dat <- long_valid %>%

group_by(patient_id, sustained_rosc) %>%

summarise(

event_avg_pi_pct = mean(pi, na.rm = TRUE),

event_avg_petco2_mmhg = mean(petco2, na.rm = TRUE),

valid_pi_count = sum(!is.na(pi)),

valid_petco2_count = sum(!is.na(petco2)),

.groups = "drop"

)

\# Group summaries

event_dat %>%

group_by(sustained_rosc) %>%

summarise(

pi_median = median(event_avg_pi_pct),

pi_q1 = quantile(event_avg_pi_pct, 0.25),

pi_q3 = quantile(event_avg_pi_pct, 0.75),

pet_median = median(event_avg_petco2_mmhg),

pet_q1 = quantile(event_avg_petco2_mmhg, 0.25),

pet_q3 = quantile(event_avg_petco2_mmhg, 0.75)

)

wilcox.test(event_avg_pi_pct ~ sustained_rosc, data = event_dat, exact = FALSE)

wilcox.test(event_avg_petco2_mmhg ~ sustained_rosc, data = event_dat, exact = FALSE)

**8. Primary ROC analyses and combined logistic model**

event_model_dat <- dat %>%

select(patient_id, sustained_rosc, age_years, time_to_cpr_min,

no_flow_time_min, epinephrine_dose_group, cpr_duration_group,

initial_rhythm_code, converted_rhythm_code,

sodium_bicarbonate_ml, defibrillation_code) %>%

left_join(event_dat, by = c("patient_id", "sustained_rosc"))

roc_pi <- roc(

event_model_dat$sustained_rosc,

event_model_dat$event_avg_pi_pct,

ci = TRUE,

direction = "<"

)

roc_pet <- roc(

event_model_dat$sustained_rosc,

event_model_dat$event_avg_petco2_mmhg,

ci = TRUE,

direction = "<"

)

combined_fit <- glm(

sustained_rosc ~ event_avg_pi_pct + event_avg_petco2_mmhg,

family = binomial(),

data = event_model_dat

)

event_model_dat$combined_prob <- predict(

combined_fit,

type = "response"

)

roc_combined <- roc(

event_model_dat$sustained_rosc,

event_model_dat$combined_prob,

ci = TRUE,

direction = "<"

)

auc(roc_pi); ci.auc(roc_pi)

auc(roc_pet); ci.auc(roc_pet)

auc(roc_combined); ci.auc(roc_combined)

\# Optimal Youden cutoffs

coords(roc_pi, "best", best.method = "youden",

ret = c("threshold", "sensitivity", "specificity"))

coords(roc_pet, "best", best.method = "youden",

ret = c("threshold", "sensitivity", "specificity"))

coords(roc_combined, "best", best.method = "youden",

ret = c("threshold", "sensitivity", "specificity"))

summary(combined_fit)

**9. DeLong pairwise comparisons with 95% CI for AUC differences**

\# Paired DeLong tests

roc.test(roc_pi, roc_pet, method = "delong", paired = TRUE)

roc.test(roc_pi, roc_combined, method = "delong", paired = TRUE)

roc.test(roc_pet, roc_combined, method = "delong", paired = TRUE)

\# 95% CI for paired AUC difference using DeLong covariance

auc_diff_ci <- function(roc1, roc2) {

a1 <- as.numeric(auc(roc1))

a2 <- as.numeric(auc(roc2))

v1 <- as.numeric(var(roc1, method = "delong"))

v2 <- as.numeric(var(roc2, method = "delong"))

cv <- as.numeric(cov(roc1, roc2, method = "delong"))

diff <- a1 - a2

se <- sqrt(v1 + v2 - 2 * cv)

z <- diff / se

p <- 2 * pnorm(-abs(z))

data.frame(

auc_difference = diff,

ci_lower = diff - 1.96 * se,

ci_upper = diff + 1.96 * se,

z = z,

p_value = p

)

}

auc_diff_ci(roc_pi, roc_pet)

auc_diff_ci(roc_pi, roc_combined)

auc_diff_ci(roc_pet, roc_combined)

**10. ROC figure**

png("ROC_event_average_models.png",

width = 1800, height = 1600, res = 250)

plot(roc_pi,

lwd = 2,

legacy.axes = TRUE,

main = "ROC curves for predicting sustained ROSC")

plot(roc_pet, add = TRUE, lwd = 2, lty = 2)

plot(roc_combined, add = TRUE, lwd = 2, lty = 3)

legend(

"bottomright",

legend = c(

paste0("Event-average PI (AUC=", round(auc(roc_pi), 3), ")"),

paste0("Event-average PETCO2 (AUC=", round(auc(roc_pet), 3), ")"),

paste0("PI + PETCO2 (AUC=", round(auc(roc_combined), 3), ")")

),

lwd = 2,

lty = c(1, 2, 3),

bty = "n"

)

dev.off()

**11. Bootstrap internal validation of the combined model**

The following code estimates optimism in AUC and Brier score by fitting the same two-predictor combined model in 2,000 bootstrap samples and evaluating each bootstrap-fitted model both in the bootstrap sample and in the original cohort.

set.seed(20260819)

dboot <- event_model_dat %>%

select(sustained_rosc, event_avg_pi_pct, event_avg_petco2_mmhg) %>%

na.omit()

apparent_fit <- glm(

sustained_rosc ~ event_avg_pi_pct + event_avg_petco2_mmhg,

family = binomial(),

data = dboot

)

p_app <- predict(apparent_fit, type = "response")

auc_app <- as.numeric(

auc(roc(dboot$sustained_rosc, p_app, direction = "<"))

)

brier_app <- mean((dboot$sustained_rosc - p_app)^2)

B <- 2000

auc_optimism <- numeric(B)

brier_optimism <- numeric(B)

cal_intercept_test <- numeric(B)

cal_slope_test <- numeric(B)

for (b in seq_len(B)) {

idx <- sample(seq_len(nrow(dboot)), replace = TRUE)

db <- dboot[idx, ]

fit_b <- glm(

sustained_rosc ~ event_avg_pi_pct + event_avg_petco2_mmhg,

family = binomial(),

data = db

)

p_train <- predict(fit_b, newdata = db, type = "response")

p_test <- predict(fit_b, newdata = dboot, type = "response")

auc_train <- as.numeric(

auc(roc(db$sustained_rosc, p_train, direction = "<", quiet = TRUE))

)

auc_test <- as.numeric(

auc(roc(dboot$sustained_rosc, p_test, direction = "<", quiet = TRUE))

)

brier_train <- mean((db$sustained_rosc - p_train)^2)

brier_test <- mean((dboot$sustained_rosc - p_test)^2)

auc_optimism[b] <- auc_train - auc_test

brier_optimism[b] <- brier_test - brier_train

lp_test <- qlogis(pmin(pmax(p_test, 1e-8), 1 - 1e-8))

cal_b <- glm(dboot$sustained_rosc ~ lp_test, family = binomial())

cal_intercept_test[b] <- coef(cal_b)[1]

cal_slope_test[b] <- coef(cal_b)[2]

}

optimism_corrected_auc <-

auc_app - mean(auc_optimism, na.rm = TRUE)

optimism_corrected_brier <-

brier_app + mean(brier_optimism, na.rm = TRUE)

bootstrap_calibration_intercept <-

mean(cal_intercept_test, na.rm = TRUE)

bootstrap_calibration_slope <-

mean(cal_slope_test, na.rm = TRUE)

c(

apparent_auc = auc_app,

corrected_auc = optimism_corrected_auc,

apparent_brier = brier_app,

corrected_brier = optimism_corrected_brier,

calibration_intercept = bootstrap_calibration_intercept,

calibration_slope = bootstrap_calibration_slope

)

**12. Calibration plot**

calibration_data <- event_model_dat %>%

select(sustained_rosc, combined_prob) %>%

na.omit() %>%

mutate(

decile = ntile(combined_prob, 10)

) %>%

group_by(decile) %>%

summarise(

predicted = mean(combined_prob),

observed = mean(sustained_rosc),

n = n(),

.groups = "drop"

)

ggplot(calibration_data,

aes(x = predicted, y = observed)) +

geom_abline(intercept = 0, slope = 1, linetype = 2) +

geom_line() +

geom_point(size = 2.5) +

coord_equal(xlim = c(0, 1), ylim = c(0, 1)) +

labs(

x = "Predicted probability of ROSC",

y = "Observed proportion with ROSC",

title = "Calibration of the combined PI + PETCO2 model"

) +

theme_classic()

**13. Parsimonious clinically interpretable adjusted model**

This model uses baseline variables available in the analysis file plus the event-average biomarkers. It is preferable for interpretation of the independent biomarker associations because it avoids conditioning on resuscitation variables that may themselves be consequences of time to ROSC.

main_adjusted_model <- glm(

sustained_rosc ~ age_years +

no_flow_time_min +

factor(initial_rhythm_code) +

event_avg_pi_pct +

event_avg_petco2_mmhg,

family = binomial(),

data = event_model_dat

)

summary(main_adjusted_model)

\# ORs and 95% CIs

exp(cbind(

OR = coef(main_adjusted_model),

confint(main_adjusted_model)

))

\# PI effect expressed per 0.1% rather than per 1%

beta_pi <- coef(main_adjusted_model)["event_avg_pi_pct"]

ci_pi <- confint(main_adjusted_model)["event_avg_pi_pct", ]

c(

OR_per_0.1pct = exp(beta_pi * 0.1),

CI_low = exp(ci_pi[1] * 0.1),

CI_high = exp(ci_pi[2] * 0.1)

)

**14. Exploratory fully adjusted model (Table 6 sensitivity model)**

This model deliberately includes all available baseline and resuscitation-related variables requested for the full sensitivity analysis. CPR duration and cumulative epinephrine dose may be influenced by whether and when ROSC occurs; therefore, coefficients from this model should be described as exploratory associations rather than causal effects.

full_model_dat <- event_model_dat %>%

mutate(

\# Scale PI to report OR per 0.1% increase

event_avg_pi_per_0.1 = event_avg_pi_pct / 0.1,

\# Scale bicarbonate to report OR per 125 mL

sodium_bicarbonate_per125 = sodium_bicarbonate_ml / 125,

epinephrine_dose_group = factor(epinephrine_dose_group),

cpr_duration_group = factor(cpr_duration_group),

initial_rhythm_code = factor(initial_rhythm_code),

converted_rhythm_code = factor(converted_rhythm_code),

defibrillation_code = factor(defibrillation_code)

)

full_adjusted_model <- glm(

sustained_rosc ~

age_years +

time_to_cpr_min +

no_flow_time_min +

event_avg_pi_per_0.1 +

event_avg_petco2_mmhg +

epinephrine_dose_group +

cpr_duration_group +

initial_rhythm_code +

converted_rhythm_code +

sodium_bicarbonate_per125 +

defibrillation_code,

family = binomial(),

data = full_model_dat

)

summary(full_adjusted_model)

\# Complete Table 6: beta, SE, adjusted OR, 95% CI, P

coef_tab <- summary(full_adjusted_model)$coefficients

ci_tab <- confint.default(full_adjusted_model)

table6 <- data.frame(

variable = rownames(coef_tab),

beta = coef_tab[, "Estimate"],

se = coef_tab[, "Std. Error"],

adjusted_OR = exp(coef_tab[, "Estimate"]),

CI_lower = exp(ci_tab[, 1]),

CI_upper = exp(ci_tab[, 2]),

p_value = coef_tab[, "Pr(>|z|)"],

row.names = NULL

)

table6

**15. Multicollinearity and linearity in the logit**

\# VIF: for factor predictors, car::vif() may return GVIF.

vif(full_adjusted_model)

\# For continuous predictors, assess x*log(x) terms one at a time.

\# Variables must be >0 for log(); add a small constant if zeros are possible.

linearity_dat <- full_model_dat %>%

mutate(

age_xlogx = age_years * log(age_years),

noflow_xlogx = (no_flow_time_min + 0.01) *

log(no_flow_time_min + 0.01),

pi_xlogx = (event_avg_pi_pct + 0.001) *

log(event_avg_pi_pct + 0.001),

pet_xlogx = event_avg_petco2_mmhg *

log(event_avg_petco2_mmhg)

)

\# Example test for PI

fit_pi_linear <- glm(

sustained_rosc ~ age_years + no_flow_time_min +

event_avg_pi_pct + event_avg_petco2_mmhg,

family = binomial(),

data = linearity_dat

)

fit_pi_nonlin <- update(

fit_pi_linear,

. ~ . + pi_xlogx

)

anova(fit_pi_linear, fit_pi_nonlin, test = "LRT")

**16. Exploratory landmark ROC sensitivity analyses**

landmark_analysis <- function(data, landmark) {

pi_var <- paste0("pi_", landmark, "min_pct")

pet_var <- paste0("petco2_", landmark, "min_mmhg")

d <- data %>%

filter(cpr_duration_min >= landmark) %>%

select(

sustained_rosc,

all_of(pi_var),

all_of(pet_var)

) %>%

na.omit()

names(d)[2:3] <- c("pi", "petco2")

fit <- glm(

sustained_rosc ~ pi + petco2,

family = binomial(),

data = d

)

d$combined_prob <- predict(fit, type = "response")

r_pi <- roc(d$sustained_rosc, d$pi, direction = "<", quiet = TRUE)

r_pet <- roc(d$sustained_rosc, d$petco2, direction = "<", quiet = TRUE)

r_comb <- roc(d$sustained_rosc, d$combined_prob,

direction = "<", quiet = TRUE)

data.frame(

landmark_min = landmark,

n_at_risk = nrow(d),

subsequent_rosc = sum(d$sustained_rosc == 1),

non_rosc = sum(d$sustained_rosc == 0),

pi_auc = as.numeric(auc(r_pi)),

petco2_auc = as.numeric(auc(r_pet)),

combined_auc = as.numeric(auc(r_comb)),

combined_ci_low = as.numeric(ci.auc(r_comb)[1]),

combined_ci_high = as.numeric(ci.auc(r_comb)[3])

)

}

landmark_results <- bind_rows(

landmark_analysis(dat, 5),

landmark_analysis(dat, 10),

landmark_analysis(dat, 15),

landmark_analysis(dat, 20)

)

landmark_results
